# Physical activity volume and intensity distribution in relation to bone, lean and fat mass in children

**DOI:** 10.1111/sms.14255

**Published:** 2022-11-17

**Authors:** Annie M. Skinner, Dimitris Vlachopoulos, Alan R. Barker, Sarah A. Moore, Alex V. Rowlands, Sonja Soininen, Eero A. Haapala, Juuso Väistö, Kate Westgate, Soren Brage, Timo A. Lakka

**Affiliations:** ^1^ Children's Health and Exercise Research Centre University of Exeter Exeter UK; ^2^ Institute of Biomedicine University of Eastern Finland Kuopio Finland; ^3^ School of Health and Human Performance Dalhousie University Halifax Canada; ^4^ Assessment of Movement Behaviours Group (AMBer), Leicester Lifestyle and Health Research Group, Diabetes Research Centre University of Leicester Leicester UK; ^5^ NIHR Leicester Biomedical Research Centre Leicester UK; ^6^ Division of Health Sciences, Alliance for Research in Exercise, Nutrition and Activity (ARENA), Sansom Institute for Health Research University of South Australia Adelaide South Australia Australia; ^7^ Social and Health Center Varkaus Finland; ^8^ Faculty of Sport and Health Sciences University of Jyväskylä Jyväskylä Finland; ^9^ MRC Epidemiology Unit University of Cambridge Cambridge UK; ^10^ Department of Clinical Physiology and Nuclear Medicine Kuopio University Hospital Kuopio Finland; ^11^ Foundation for Research in Health Exercise and Nutrition Kuopio Research Institute of Exercise Medicine Kuopio Finland

**Keywords:** accelerometry, adiposity, bone mineral content, DXA, intensity gradient, pediatrics

## Abstract

Considering physical activity (PA) volume and intensity may provide novel insights into the relationships of PA with bone, lean, and fat mass. This study aimed to assess the associations of PA volume, PA intensity distribution, including moderate‐to‐vigorous PA (MVPA) with total‐body‐less‐head bone mineral content (BMC), lean, and fat mass in children. A population sample of 290 Finnish children (158 females) aged 9–11 years from the Physical Activity and Nutrition in Children (PANIC) Study was studied. PA, including MVPA, was assessed with a combined heart rate and movement sensor, and the uniaxial acceleration was used to calculate average‐acceleration (a proxy metric for PA volume) and intensity‐gradient (reflective of PA intensity distribution). Linear regression analyzed the associations of PA volume, PA intensity and MVPA with BMC, lean mass, and fat mass assessed by dual‐energy X‐ray absorptiometry. PA volume was positively associated with BMC in females (unstandardised regression coefficient [*ß*] = 0.26) and males (*ß* = 0.47), and positively associated with lean (*ß* = 7.33) and negatively associated with fat mass in males (*ß* = −20.62). PA intensity was negatively associated with BMC in males (*ß* = −0.13). MVPA was positively associated with lean mass in females and males (*ß* = 0.007 to 0.012), and negatively associated with fat mass in females and males (*ß* = −0.030 to −0.029). PA volume may be important for improving BMC in females and males, and increasing lean and reducing fat mass in males, whereas MVPA may be important for favorable lean and fat outcomes in both sexes.

## 1 INTRODUCTION

Physical activity (PA) is positively associated with bone mineral content (BMC) and lean mass and inversely associated with fat mass in children and adolescents.^1^, ^2^ PA increases BMC by increasing mechanical loads on bones, and the skeleton adapts in response to these strains.^3^ PA may also indirectly influence BMC via lean and fat mass, as greater lean and fat mass increase mechanical loading on bones.^3^ During childhood and adolescence, BMC, lean mass, and fat mass increase with linear growth, with the amount of bone accrued during growth potentially determining the risk of osteoporosis in later life.^4^, ^5^ BMC tracks throughout childhood and adolescence, and as such, the interrelationships between BMC, body composition and PA in pre‐ and early‐puberty are of particular interest.^4^, ^5^

Few studies have considered bone, muscle, and fat outcomes together when investigating the association between PA and BMC. In children aged 11 years, device‐measured moderate‐to‐vigorous PA (MVPA) was positively associated with total‐body‐less‐head (TBLH) BMC, controlling for lean and fat mass, though this association was not significant when only controlling for lean mass.^6^ Females and males^*^ * with greater levels of MVPA from age 5 to 17 years had greater leg lean mass and greater proximal femur areal bone mineral density (aBMD) at age 17, with 29%–49% of the relationship between MVPA and aBMD mediated via lean mass, though fat mass was not considered.^2^ Although these studies support the importance of considering body composition when investigating the association between PA and bone mass, previous research has focused on MVPA as a summary measure of PA intensity, which is related to energy expenditure.^2^, ^6^ Applying cut‐points to categorize PA intensity condenses the PA intensity continuum into broad categories, validated against oxygen consumption, which may not be relevant for muscle and bone strengthening activities.^2^, ^7^, ^8^ Further, as movement is accumulated across an intensity continuum, rather than focusing on specific intensities of activity, the whole intensity spectrum should be considered when examining the relationships of PA with BMC, lean mass, and fat mass.^7^

To address the limitations of applying cut‐points to PA data, Rowlands et al.^9^ proposed using two accelerometer metrics to capture the volume and intensity distribution of the PA profile. PA volume is reflected in the average‐acceleration and intensity distribution and can be characterized by the gradient of the relationship between intensity and time accumulated at that intensity.^9^ In adolescents and young adults, PA volume and intensity distribution were positively associated with TBLH BMC, indicating that accumulating PA volume at any intensity, or increasing intensity without increasing volume, could be beneficial for BMC.^7^ However, the associations of PA volume and intensity distribution with BMC, lean mass, and fat mass, compared with the traditional approach of summarizing MVPA based on energy expenditure, in pre‐ and early‐pubertal children remain unknown.

This study aimed to: (1) assess the associations of PA volume (average‐acceleration) and intensity distribution (intensity‐gradient) with TBLH BMC, lean mass, and fat mass in a population sample of pre‐ and early‐pubertal children aged 9–11 years; (2) repeat the analysis with MVPA as the PA exposure variable, to check whether the findings differ based on accelerometer metrics used; and (3) apply translational metrics to illustrate the profile of the PA volume and intensity distribution associated with improved BMC and lean mass, and reduced fat mass in this cohort.

## 2 MATERIALS AND METHODS

### 2.1 Study design and participants

This study used cross‐sectional data from the 2‐year follow‐up of the Physical Activity and Nutrition in Children (PANIC) Study, an 8‐year controlled lifestyle intervention study in a population sample of Finnish children (ClinicalTrials.gov registration number NCT01803776) that continues as a follow‐up study.^10^ Children aged 6–9 years who were registered for the first grade in public school in the city of Kuopio, Finland were invited to participate in baseline examinations between 2007 and 2009. Children were eligible to participate if they had no disability that could prevent their participation in the assessments or the intervention and if both the child and their custodian were able to communicate in Finnish to fill out the questionnaires and participate in the intervention. Of the 736 children invited to participate at baseline, 512 attended the baseline examinations, of which 504 were included in the final baseline sample. At 2‐year follow‐up, 438 attended examinations. Additional information about the PANIC study is presented elsewhere.^10^ Given the importance of the pre‐ and early‐pubertal period for bone development, the 2‐year follow‐up data were used in these analyses, when children were aged 9–11 years, in order to capture this potentially critical period for bone accrual.^4^, ^5^

**FIGURE 1:**
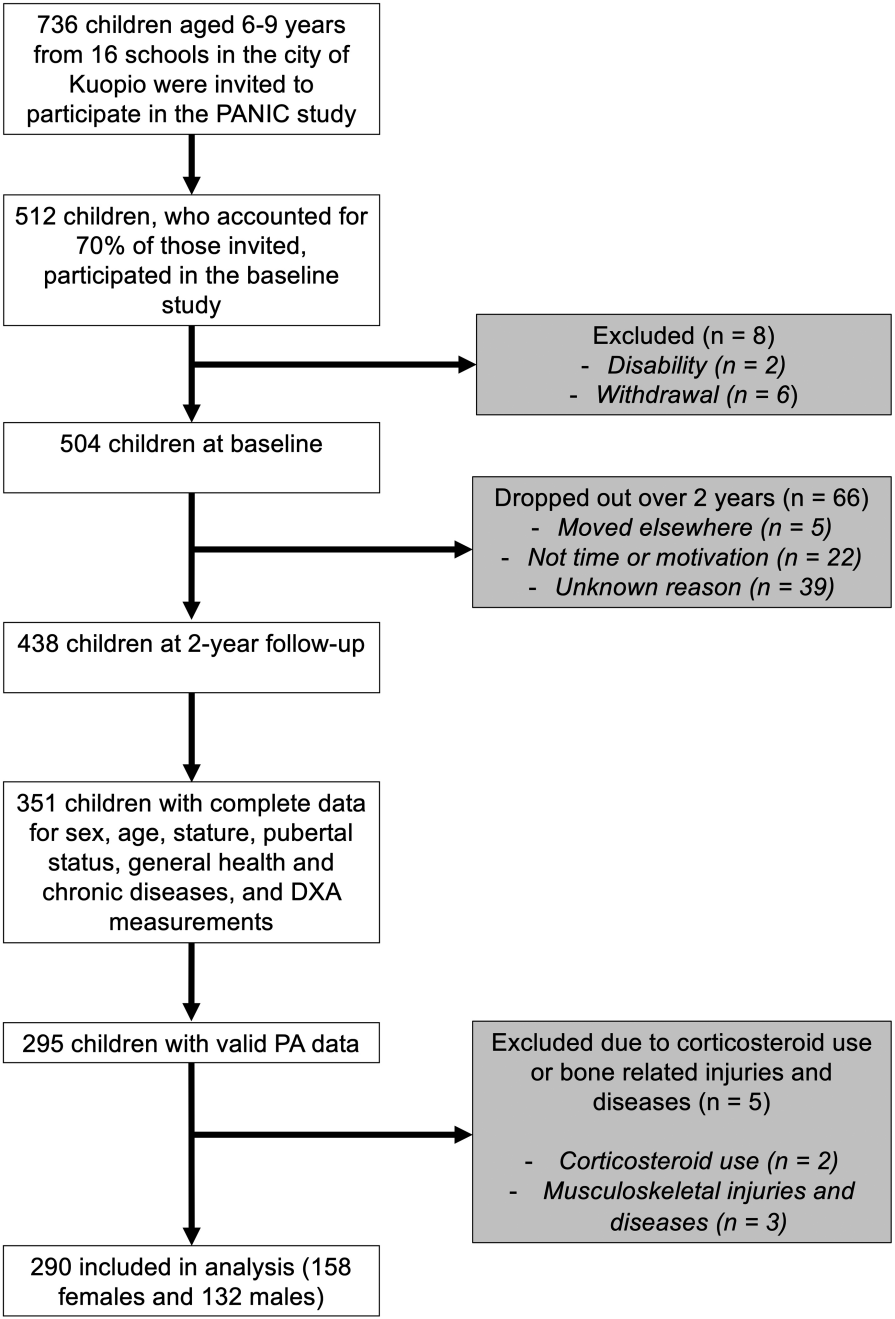
Participant flow chart. DXA, dual‐energy X‐ray absorptiometry; PA, physical activity; PANIC, physical activity and nutrition in children.

For the present analyses, we excluded children who used oral corticosteroids, as they could influence BMC,^5^ and children with musculoskeletal injuries and diseases. Complete and valid data on the main variables used in the present analyses were available for 290 children (158 females). Of these children, 99% were Caucasian. The children included in these analyses did not differ in age, stature, pubertal status, weight status, TBLH BMC, lean mass, or fat mass to the children who did not have complete data (Appendix S1, Table S1). Inclusion and exclusion criteria are displayed in Figure 1. The study protocol was approved by the Research Ethics Committee of the Hospital District of Northern Savo, and the study was conducted according to the ethical guidelines of the Declaration of Helsinki. The parents or caregivers of the children provided their written informed consent, and the children provided their assent to participation.

### 2.2 Assessment of general health and pubertal status

General health was assessed by a questionnaire including items on children's chronic diseases and allergies diagnosed by a physician and information on children's medication use, completed by caregivers. Pubertal status was assessed by a research physician and determined as breast development at Tanner stage ≥2 for females and testicular volume ≥4 ml assessed by an orchidometer for males.^11^

### 2.3 Anthropometry

Stature was measured three times to an accuracy of 0.1 cm. Body weight was measured twice using the InBody 720 bioelectrical impedance analysis (BIA) device (Biospace) to an accuracy of 0.1 kg, with children in a fasted state, having emptied the bladder. For stature and body weight, the mean of the values was used in analyses. Body mass index (BMI) (kg/m^2^) was calculated, and the BMI cut‐offs were applied to classify children as thin, normal weight, or living with overweight or obesity as it related to their weight status.^12^

### 2.4 Assessment of bone mineral content and body composition

TBLH BMC (kg), total lean mass (kg), and total fat mass (kg) were measured using the Lunar Prodigy Advance dual‐energy X‐ray absorptiometry (DXA) device (GE Medical Systems) and the Encore software, Version 10.51.006 (GE Company). Lower‐limb and upper‐limb BMC, lean mass, and fat mass were automatically defined from whole body DXA scans using Encore software (GE Company), and a mean of both sides was used. TBLH BMC, lower‐limb BMC, upper‐limb BMC were used as the bone outcomes of interest, as evidence indicates that for pre‐ and early‐pubertal children BMC is a more accurate and reliable measure than aBMD.^13^ DXA provides valid and reliable data on BMC and body composition in children (coefficient of variation = 0.01%–4.37%).^14^

### 2.5 Assessment of physical activity

Physical activity was assessed using Actiheart (CamNtech Ltd), a combined heart rate and movement sensor,^8^, ^15^ using methods described previously in this cohort.^16^ Heart rate and acceleration were recorded in 60‐s epochs. Participants were instructed to wear the monitor continuously across the day for a minimum of four consecutive days, although some children wore the monitor for up to 9 days. As PA patterns differ between weekdays and weekends, the wear period was scheduled to include an entire weekend.^17^ MVPA was modeled from the combined sensing signal using a branched equation framework.^16^, ^18^ The acceleration data were summarized in counts, and converted to the International System of Units unit of m/s^2^ by multiplying Actiheart counts by 0.003.^15^ Non‐wear time was classified as zero‐acceleration lasting >90 min combined with non‐physiological heart rate.^16^ Diurnal imbalance in non‐wear was minimized when summarizing the data to reduce bias and error as previously described, and PA data were expressed as the fraction of time spent at a given intensity to account for differences in wear time.^16^, ^19^ A valid PA measurement was defined as ≥48 h of good‐quality data with ≥32 h of weekday data and ≥16 h of weekend data as well as ≥12 h of morning, noon, afternoon, and evening wear time to protect against bias from over‐representation from specific times of day and to optimize the diurnal bias minimisation procedure.^16^, ^19^

For the present analysis, we primarily used the uniaxial acceleration signal as the PA exposure, as mechanical loading is more relevant to bone than PA energy expenditure.^20^ The combined sensing data were used to assess MVPA based on energy expenditure, as is traditionally used in PA research.^21^, ^22^ MVPA was categorized as time spent ≥4 metabolic equivalents, with 3.5 ml O_2_/min/kg used to define resting metabolic rate.^21^, ^22^

#### 2.5.1 Intensity distribution

**FIGURE 2:**
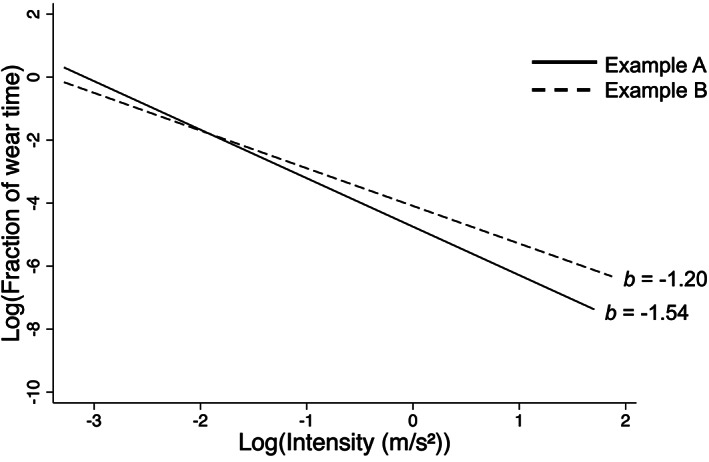
Example of the fitted regression line of log‐transformed intensity and fraction of wear time. These examples are from a 24‐h protocol and adjusted for diurnal imbalance in non‐wear, based on two participant's intensity distributions. Participant A (solid line) spent less of their total wear time at a high intensity of physical activity, resulting in a lower intensity‐gradient (*b* = −1.54) compared to participant B (dotted line) who has a higher intensity‐gradient (*b* = −1.20).

The process for calculating the intensity‐gradient is provided in Appendix S2 and is based on the method previously described elsewhere.^9^ Briefly, the acceleration signal was summarized as a fraction of wear time spent in 25 acceleration incremental bands (m/s^2^) across the movement intensity continuum. The negative curvilinear relationship between intensity and time accumulated at that intensity was described with the gradient from a natural log–log regression, providing a measure of a participant's PA intensity distribution (Figure 2). The *R* ^*2*^ (indicative of goodness of fit of the linear model), gradient (*b*_1_), 95% confidence intervals (CI) for the gradient, and intercept (*b*_0_) of the regression equation were recorded for each participant.^9^

#### 2.5.2 Average‐acceleration

The average‐acceleration is calculated as the mean acceleration across wear time, adjusted for diurnal imbalance in non‐wear, providing a proxy for the daily volume of PA.^9^

#### 2.5.3 Translational Metrics

Translational metrics were calculated as the intensity above which a child's most active 2, 5, 10, 15, 30, 60, 120, 240, and 480 min (MX metrics, whereby *X* = time in minutes) were accumulated. These metrics provide an illustration of how PA is accumulated.^23^ Levels of acceleration associated with different activities were applied to allow interpretation of MX metrics. Activities such as skipping, running, and soccer were characterized as acceleration >4 m/s^2^, based on data in children aged 8 years,^20^ 13 years,^22^ and 6–16 years.^24^ Brisk walking (5.2 km/h) was characterized as acceleration ~1.5 m/s^2^, and slow walking (3.2 km/h) was characterized as acceleration ~0.75 m/s^2^, based on data in children aged 13 years.^22^

### 2.6 Statistical analysis

Analyses were performed with Stata/SE for Mac software, Version 16.1 (StataCorp LLC), and radar plots created in R software.^25^ As there were no differences between the included and excluded children in terms of age, stature, body weight, BMI categories, pubertal status, and TBLH BMC, lean, and fat mass, we proceeded with a complete‐case analysis (Appendix S1, Table S1). The means and standard deviations (SDs) or the medians and interquartile ranges (IQR) were calculated for the total study sample and stratified by sex. Independent samples *t*‐tests, Mann–Whitney *U* tests, and Fisher's exact tests were used to test for sex differences in participant characteristics. We stratified all further analyses by sex, based on the biological differences between females and males in the studied age group.^4^

Linear regression was used to assess the associations of average‐acceleration and intensity‐gradient with TBLH BMC, lean mass, and fat mass. Activity variables were mean‐centred for analysis. Model 1 included the activity variable (average‐acceleration or intensity‐gradient) adjusted for age, stature, pubertal status, and accelerometer wear time. Adjusting for age, stature and pubertal status is recommended when examining BMC in pediatric populations.^26^ Model 2 included additional adjustment for the alternate activity metric (average‐acceleration or intensity‐gradient), and Model 3 additionally included the interaction term for average‐acceleration by intensity‐gradient.^26^ For BMC as the outcome, Models 4 and 5 included additional adjustment for lean and fat mass, respectively, and Model 6 included adjustment for lean and fat mass.^6^ For lean mass as the outcome, the final model (Model 5) included adjustment for fat mass, and for fat mass at the outcome, the final model (Model 4) included adjustment for lean mass. Analysis was repeated with MVPA as the PA exposure variable. Although the PANIC study included a lifestyle intervention, there were no differences between the intervention and control group in terms of TBLH BMC, lower‐limb BMC, upper‐limb BMC, lean mass, fat mass, average‐acceleration, intensity‐gradient, and MVPA. We therefore did not adjust for the intervention in our analyses. Unstandardised regression coefficients (*ß*), their 95% CIs and *p*‐values were reported. The alpha was set as 0.05.

To present descriptive MX metrics, the females and males were stratified into four groups based on the sex‐ specific means for average‐acceleration and intensity‐gradient. Children were split into (1) low‐volume (average‐acceleration < mean) and low‐intensity (intensity‐gradient < mean), (2) low‐volume (average‐acceleration < mean) and high‐intensity (intensity‐gradient ≥ mean), (3) high‐volume (average‐acceleration ≥ mean) and low‐intensity (intensity‐gradient < mean), and (4) high‐volume (average‐acceleration ≥ mean) and high‐intensity (intensity‐gradient ≥ mean). This allows the description of PA patterns in children with a similar volume but varying intensity distribution, and vice versa. The mean and standard error of the group MX metrics based on the raw data and standardized based on the sex‐specific mean were visualized on radar plots, using an openly accessible R script.^23^, ^27^ To demonstrate how PA could be accumulated for a 1 SD greater average‐acceleration, we applied the calculation by Rowlands and colleagues, assuming that the introduced activity would replace time spent at the average‐acceleration: 1440 × (SD of average‐acceleration)/(acceleration associated with a specific activity − average‐acceleration) (Appendix S3).^9^

## 3 RESULTS

### 3.1 Descriptive characteristics

In this study, females were younger, shorter, and lighter, with lower levels of BMC, lean mass, MVPA, and lower average‐acceleration compared to males (Table 1). The proportion of pubertal children was greater in females than males, whereas more males were pre‐pubertal. The correlations (data not shown) between the average‐acceleration and intensity‐gradient was 0.66 (*p* < 0.001), between the average‐acceleration and MVPA was 0.62 (*p* < 0.001), and between the intensity‐gradient with MVPA was 0.39 (*p* < 0.001).

**TABLE 1:** Descriptive characteristics of children

	Total (*n* = 290)	Females (*n* = 158)	Males (*n* = 132)	*p*‐Value for sex difference
Age (years)	9.8 (0.4)	9.7 (0.4)	9.8 (0.5)	**0.02**
Stature (cm)	140.8 (6.1)	140.1 (6.3)	141.7 (5.7)	**0.03**
Body weight (kg)	32.9 (29.5 to 38.7)	32.2 (28.8 to 35.9)	34.3 (30.1 to 39.5)	**0.01**
BMI‐SDS	−0.2 (1)	−0.2 (1)	−0.1 (1.1)	0.34
Pubertal status (*n*, %)				
Prepubertal	218 (75.2)	103 (65.2)	115 (87.1)	**<0.001**
Pubertal	72 (24.8)	55 (34.8)	17 (12.9)	
IOTF definition (*n*, %)				
Thin	31 (10.7)	15 (9.5)	16 (12.1)	0.52
Normal weight	216 (74.5)	123 (77.9)	93 (70.5)	
Overweight	33 (11.4)	16 (10.1)	17 (12.9)	
Obese	10 (3.4)	4 (2.5)	6 (4.5)	
TBLH BMC (kg)	0.9 (0.2)	0.9 (0.2)	1 (0.2)	**0.002**
TBLH lean mass (kg)	21.8 (2.9)	20.8 (2.7)	23.0 (2.7)	**<0.001**
TBLH fat mass (kg)	6.7 (4.4 to 10.3)	6.7 (5 to 10.9)	6.7 (3.8 to 10)	0.16
MVPA (min/day)	89.7 (57.1 to 127.3)	71.5 (51.3 to 99.2)	115.2 (78.9 to 161.9)	**<0.001**
PA guidelines^a^ (*n*, %)				
< mean 60 min MVPA/day	77 (26.7)	54 (34.4)	23 (17.6)	**0.001**
≥ mean 60 min MVPA/day	211 (73.3)	103 (65.6)	108 (82.4)	
Average‐acceleration (m/s^2^)	0.20 (0.06)	0.19 (0.06)	0.22 (0.07)	**0.002**
Intensity‐gradient	−1.67 (0.16)	−1.69 (0.14)	−1.65 (0.18)	0.07
Wear time (h)	103.2 (14.8)	103.0 (13.5)	103.5 (16.3)	0.79
MX metrics (m/s^2^)				
M2	4.13 (3.50 to 4.75)	4.00 (3.50 to 4.75)	4.25 (3.50 to 5.00)	0.53
M5	3.50 (2.75 to 4.00)	3.25 (2.75 to 4.00)	3.50 (3.00 to 4.13)	0.21
M10	2.75 (2.25 to 3.25)	2.75 (2.25 to 3.25)	2.75 (2.50 to 3.50)	0.09
M15	2.50 (2.00 to 3.00)	2.50 (2.00 to 2.75)	2.50 (2.25 to 3.00)	0.06
M30	2.00 (1.50 to 2.25)	1.75 (1.50 to 2.25)	2.00 (1.75 to 2.50)	**0.005**
M60	1.25 (1.00 to 1.50)	1.25 (1.00 to 1.50)	1.50 (1.25 to 1.75)	**<0.001**
M120	0.75 (0.75 to 1.00)	0.75 (0.50 to 1.00)	1.00 (0.75 to 1.00)	**<0.001**
M1/3	0.08 (0.08 to 0.25)	0.08 (0.08 to 0.25)	0.08 (0.08 to 0.25)	0.66

*Note*: The values are means (standard deviations), medians (interquartile ranges) or numbers (percentages) of children, and *p*‐values are for the differences between females and males. Differences between females and males were tested with independent samples *t* test for continuous variables with normal distributions, with Mann–Whitney *U* test for continuous variables with skewed distributions, and with Fishers exact test for categorical variables. Bold emphasis indicates statistical significance at *p* < 0.05.

Abbreviations: BMC, bone mineral content; BMI‐SDS, Body mass index standard deviation score; IOTF, International Obesity Task Force; M1/3, the intensity above which a child's most active 480 min are accumulated; M10, the intensity above which a child's most active 10 min are accumulated; M120, the intensity above which a child's most active 120 min are accumulated; M15, the intensity above which a child's most active 15 min are accumulated; M2, the intensity above which a child's most active 2 min are accumulated; M30, the intensity above which a child's most active 30 min are accumulated; M5, the intensity above which a child's most active 5 min are accumulated; M60, the intensity above which a child's most active 60 min are accumulated; MVPA, moderate to vigorous physical activity; MX, the intensity above which a child's most active X minutes are accumulated; TBLH, total body less head.

^a^ For PA guidelines, *n* = 288 (157 females) due to poor heart rate recordings for two participants preventing the application of the branched equation modeling.

### 3.2 Associations between physical activity volume (average‐acceleration), physical activity intensity distribution (intensity‐gradient) and bone mineral content

In females, average‐acceleration was positively associated with TBLH BMC (Table 2, Model 1), though this association became non‐significant after adjustment for intensity‐gradient (Model 2), the product term of intensity‐gradient by average‐acceleration (Model 3), and lean mass (Model 4). When adjusting for fat mass (Model 5), and lean and fat mass (Model 6), average‐acceleration was positively associated with TBLH BMC. Intensity‐gradient, and the product of intensity‐gradient by average‐acceleration, were not associated with TBLH BMC in females. Associations in the fully‐adjusted model (Model 6) were non‐significant in lower‐limb BMC and in upper‐limb BMC in females.

**TABLE 2:** Associations of physical activity volume (average‐acceleration) and intensity distribution (intensity‐gradient) with total body less head bone mineral content, lean mass, and fat mass (*n* = 290).

	Model 1 (adjusted for age, stature, pubertal status, and wear time)		Model 2 (Model 1 + alternate activity metric)		Model 3 (Model 2 + intensity × volume interaction)		Model 4 (Model 3 + lean mass)		Model 5 (Model 3 + fat mass)		Model 6 (Model 3 + lean mass and fat mass)	
	*ß* (95% CI)	*p*	*ß* (95% CI)	*p*	*ß* (95% CI)	*p*	*ß* (95% CI)	*p*	*ß* (95% CI)	*p*	*ß* (95% CI)	*p*
TBLH BMC												
Females												
Intensity^a^	0.092 (−0.032 to 0.216)	0.145	0.009 (−0.144 to 0.163)	0.905	0.019 (−0.137 to 0.175)	0.809	0.034 (−0.099 to 0.167)	0.614	0.041 (−0.076 to 0.157)	0.490	0.050 (−0.048 to 0.149)	0.317
Volume^b^	**0.354 (0.053 to 0.654)**	**0.021**	0.340 (−0.035 to 0.716)	0.075	0.372 (−0.013 to 0.757)	0.058	0.201 (−0.131 to 0.533)	0.233	**0.402 (0.114 to 0.689)**	**0.007**	**0.264 (0.019 to 0.510)**	**0.035**
Intensity × volume					−0.810 (−2.878 to 1.258)	0.440	−1.560 (−3.338 to 0.219)	0.085	−0.597 (−2.144 to 0.951)	0.447	−1.209 (−2.525 to 0.106)	0.071
Males												
Intensity^a^	−0.043 (−0.167 to 0.080)	0.487	−0.167 (−0.336 to 0.003)	0.054	**−0.171 (−0.340 to −0.001)**	**0.048**	**−0.164 (−0.306 to −0.022)**	**0.024**	**−0.133 (−0.259 to −0.008)**	**0.037**	**−0.134 (−0.241 to −0.027)**	**0.015**
Volume^b^	0.160 (−0.162 to 0.483)	0.327	**0.464 (0.020 to 0.908)**	**0.041**	0.389 (−0.069 to 0.848)	0.095	0.143 (−0.248 to 0.534)	0.471	**0.698 (0.354 to 1.041)**	**0.000**	**0.471 (0.170 to 0.773)**	**0.002**
Intensity × volume					0.857 (−0.491 to 2.204)	0.211	0.771 (−0.360 to 1.903)	0.180	−0.037 (−1.046 to 0.973)	0.943	0.039 (−0.825 to 0.903)	0.928
TBLH lean mass												
Females												
Intensity^a^	1.104 (−0.582 to 2.790)	0.198	−0.153 (−2.232 to 1.926)	0.884	−0.386 (−2.487 to 1.714)	0.717			−0.302 (−2.371 to 1.767)	0.774		
Volume^b^	**4.935 (0.867 to 9.003)**	**0.018**	**5.158 (0.076 to 10.241)**	**0.047**	4.398 (−0.788 to 9.584)	0.096			4.512 (−0.595 to 9.619)	0.083		
Intensity × volume					19.270 (−8.596 to 47.137)	0.174			20.103 (−7.342 to 47.549)	0.150		
Males												
Intensity^a^	1.435 (−0.148 to 3.018)	0.075	−0.151 (−2.326 to 2.025)	0.891	−0.160 (−2.346 to 2.025)	0.885			0.027 (−2.094 to 2.149)	0.980		
Volume^b^	**5.694 (1.618 to 9.770)**	**0.007**	**5.969 (0.273 to 11.664)**	**0.040**	5.794 (−0.122 to 11.710)	0.055			**7.333 (1.513 to 13.153)**	**0.014**		
Intensity × volume					2.004 (−15.377 to 19.386)	0.820			−2.457 (−19.551 to 14.637)	0.776		
TBLH fat mass												
Females												
Intensity^a^	−1.828 (−6.286 to 2.630)	0.419	−1.326 (−6.894 to 4.242)	0.639	−1.185 (−6.844 to 4.473)	0.680	−0.984 (−6.558 to 4.589)	0.728				
Volume^b^	−3.990 (−14.894 to 6.913)	0.471	−2.059 (−15.672 to 11.554)	0.765	−1.599 (−15.572 to 12.373)	0.821	−3.884 (−17.769 to 10.001)	0.581				
Intensity × volume					−11.640 (−86.720 to 63.440)	0.760	−21.651 (−96.032 to 52.729)	0.566				
Males												
Intensity^a^	**−5.017 (−9.469 to −0.566)**	**0.027**	−1.768 (−7.933 to 4.397)	0.571	−1.997 (−8.101 to 4.107)	0.518	−1.880 (−7.794 to 4.035)	0.530				
Volume^b^	**−15.450 (−27.015 to −3.885)**	**0.009**	−12.231 (−28.371 to 3.910)	0.136	−16.376 (−32.897 to 0.145)	0.052	**−20.624 (−36.872 to −4.375)**	**0.013**				
Intensity × volume					47.458 (−1.082 to 95.999)	0.055	45.989 (−1.053 to 93.030)	0.055				

*Note*: The values are unstandardised regression coefficients (*ß*), their 95% confidence intervals (CI), and *p* values from linear regression models. Model 1 included the activity variable (average‐acceleration or intensity‐gradient) adjusted for age, stature, pubertal status, and accelerometer wear time, Model 2 included additional adjustment for the alternate activity metric (average‐acceleration or intensity‐gradient), Model 3 additionally included the interaction term for average‐acceleration by intensity‐gradient. For BMC as the outcome, Model 4 included additional adjustment for lean mass, Model 5 included additional adjustment for fat mass, and Model 6 included adjustment for lean and fat mass. For lean mass as the outcome, the final model (Model 5) included adjustment for fat mass, and for fat mass at the outcome, the final model (Model 4) included adjustment for lean mass. Bold emphasis indicates statistical significance at *p* < 0.05. Activity variables were mean‐centred before entry into analysis, with interaction terms computed from the centred scores.

Abbreviations: BMC, bone mineral content; TBLH, Total body less head.

^a^ Intensity is reflected in the intensity‐gradient, calculated from data collected with a 24‐h protocol, adjusted for diurnal imbalance in non‐wear, as the regression line from log–log plot of intensity (*x*) and fraction of wear time accumulated (*y*).

^b^ Volume is reflected in the average‐acceleration across data, collected with a 24‐h protocol, adjusted for diurnal imbalance in non‐wear.

In males, average‐acceleration was not associated with TBLH BMC (Table 2) in the minimally‐adjusted model (Model 1), though this association became significant after adjustment for intensity‐gradient (Model 2). After adjustment for the product term of intensity‐gradient by average‐acceleration (Model 3), and for lean mass (Model 4) this association was non‐significant. When adjusting for fat mass (Model 5), and for lean and fat mass (Model 6), average‐acceleration was positively associated with TBLH BMC. Intensity‐gradient was not associated with TBLH BMC (Table 2) in the minimally‐adjusted model (Model 1), or after adjustment for average‐acceleration (Model 2). When adjusting for the product of intensity‐gradient by average‐acceleration (Model 3), lean mass (Model 4), fat mass (Model 5), and lean and fat mass (Model 6), intensity‐gradient was negatively associated with TBLH BMC. The product of intensity‐gradient by average‐acceleration was not associated with TBLH BMC in any model in males. Site‐specific analyses showed that in the fully‐adjusted model (Model 6), average‐acceleration was positively associated with lower‐limb BMC, and the association between intensity‐gradient with lower‐limb BMC was non‐significant. In the fully‐adjusted model (Model 6), average‐acceleration was not associated with upper‐limb BMC, and intensity‐gradient was negatively associated with upper‐limb BMC (Appendix S1, Table S3).

### 3.3 Associations between physical activity volume (average‐acceleration), physical activity intensity distribution (intensity‐gradient) and lean mass

In females, average‐acceleration was positively associated with TBLH lean mass (Table 2), and this association persisted after adjustment for intensity‐gradient (Model 2), though became non‐significant after adjustment for the product term of intensity‐gradient by average‐acceleration (Model 3), and fat mass (Model 5). Intensity‐gradient, and the product of intensity‐gradient by average‐acceleration were not associated with TBLH lean mass in any model. Associations in the upper‐limb and lower‐limb were also non‐significant in the fully‐adjusted model (Model 5; Appendix S1, Table S2).

In males, average‐acceleration was positively associated with TBLH lean mass (Table 2), and this association persisted after adjustment for intensity‐gradient (Model 2). The association became non‐significant when adjusting for the product term of intensity‐gradient by average‐acceleration (Model 3) but was significant when adjusting for fat mass (Model 5). Intensity‐gradient, and the product of intensity‐gradient by average‐acceleration were not associated with TBLH lean mass in any model. Associations in the fully‐adjusted model (Model 5) were non‐significant in lower‐limb lean mass (Appendix S1, Table S3). In the upper‐limb, average‐acceleration was positively associated with lean mass and intensity‐gradient was negatively associated with lean mass in the fully‐adjusted model (Model 5) (Appendix S1, Table S3).

### 3.4 Associations between physical activity volume (average‐acceleration), physical activity intensity distribution (intensity‐gradient) and fat mass

In females, average‐acceleration, intensity‐gradient, and the product of intensity‐gradient by average‐acceleration were not associated with TBLH fat mass in any model. This was also the case with lower‐limb and upper‐limb fat mass (Appendix S1, Table S2).

**FIGURE 3:**
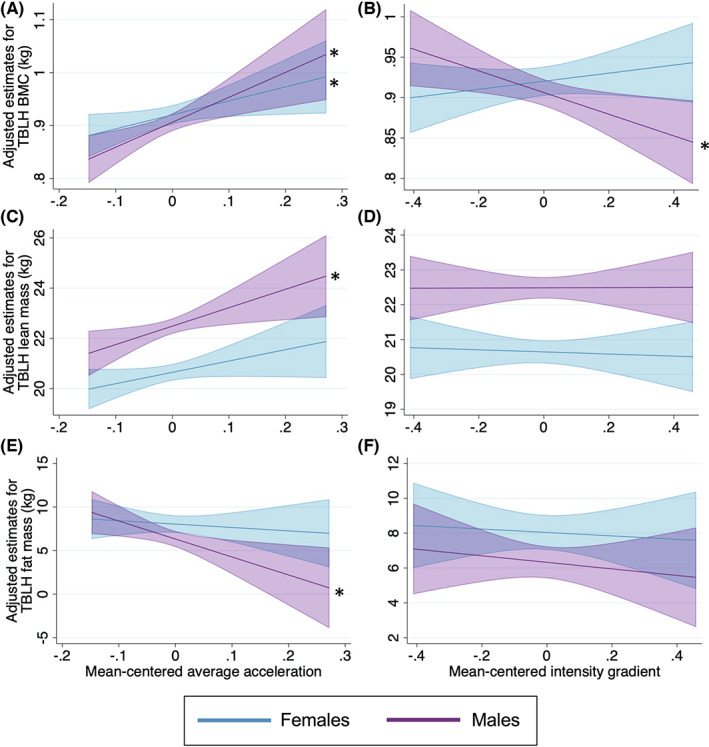
Main associations between average‐acceleration and intensity‐gradient with TBLH BMC, lean mass and fat mass. *Indicates statistical significance (*p* < 0.05). Values are predicted for a female and a male with mean levels of covariates from the fully adjusted models. All models are adjusted for age, stature, pubertal status, wear time, alternate activity metric and the product term for average acceleration by intensity gradient. A and B are additionally adjusted for lean mass and fat mass (model 6), C and D are additionally adjusted for fat mass (model 5), and E and F are additionally adjusted for lean mass (model 4). BMC, bone mineral content; TBLH, Total body less head.

In males, average‐acceleration was negatively associated with TBLH fat mass (Model 1) (Table 2). Although this association was not significant after adjustment for intensity‐gradient (Model 2), after additional adjustment for the product term of intensity‐gradient by average‐acceleration (Model 3) and for lean mass (Model 4) the negative association became significant again. Intensity‐gradient was negatively associated with TBLH fat mass (Model 1), though this association was not independent of average‐acceleration and lean mass (Model 2–4). The product of intensity‐gradient by average‐acceleration were not associated with TBLH fat mass in any model. In the lower‐limb, the associations between average‐acceleration, intensity‐gradient, and the product of intensity‐gradient by average‐acceleration were similar in terms of significance and direction to those with TBLH fat mass. In the upper‐limb, average‐acceleration was negatively associated with fat mass, and the product of intensity‐gradient by average‐acceleration was positively associated with fat mass (Appendix S1, Table S3). The adjusted main effects of average‐acceleration and intensity‐gradient with TBLH BMC, lean, and fat mass are illustrated in Figure 3.

### 3.5 Associations between MVPA with bone mineral content, lean, and fat mass

When repeating our analysis with MVPA as the exposure variable, there was no association with TBLH BMC in females or males when adjusting for lean and fat mass. MVPA was positively associated with TBLH lean mass, adjusted for fat mass, and negatively associated with TBLH fat mass, adjusted for lean mass, in females and males (Appendix S1, Table S4).

### 3.6 Translation to physical activity patterns

**FIGURE 4:**
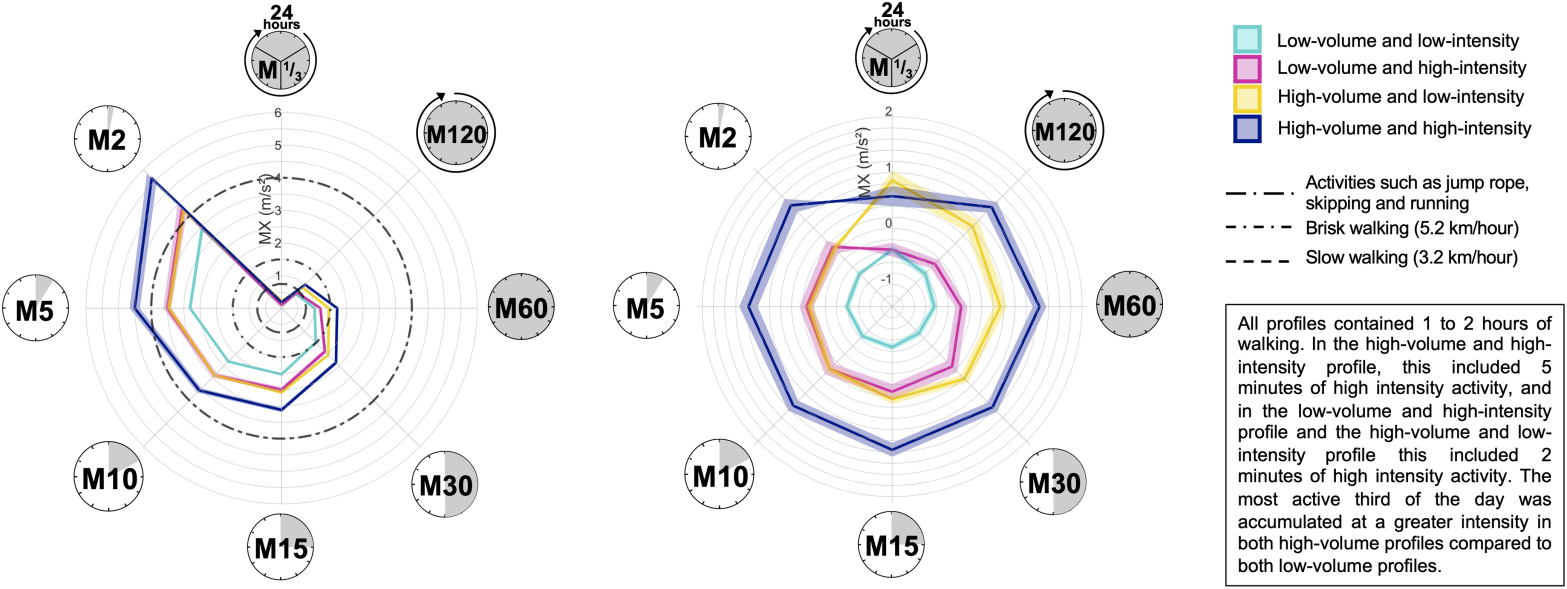
Illustration of the physical activity profile for raw (left) and standardized (right) MX metrics in females. Profiles are presented for four groups, (1) high‐volume and high‐intensity, (2) high‐volume and low‐intensity, (3) low‐volume and high‐intensity, and (4) low‐volume and low‐intensity. High‐volume was defined as average‐acceleration ≥ mean, low‐volume was defined as average‐acceleration < mean, high‐intensity was defined as intensity‐gradient ≥ mean, low‐intensity was defined as intensity‐gradient < mean. Values are the mean; error ribbons are the standard error of the mean. Standardized metrics were standardized based on the sex‐specific mean. The MX metrics show the acceleration above which a child's most active X minutes are accumulated. Each plot shows (clockwise) *M1/3*, the intensity above which a child's most active 480 min are accumulated; *M120*, the intensity above which a child's most active 120 min are accumulated; *M60*, the intensity above which a child's most active 60 min are accumulated; *M30*, the intensity above which a child's most active 30 min are accumulated; *M15*, the intensity above which a child's most active 15 min are accumulated; *M10*, the intensity above which a child's most active 10 min are accumulated; *M5*, the intensity above which a child's most active 5 min are accumulated; *M2*, the intensity above which a child's most active 2 min are accumulated.

**FIGURE 5:**
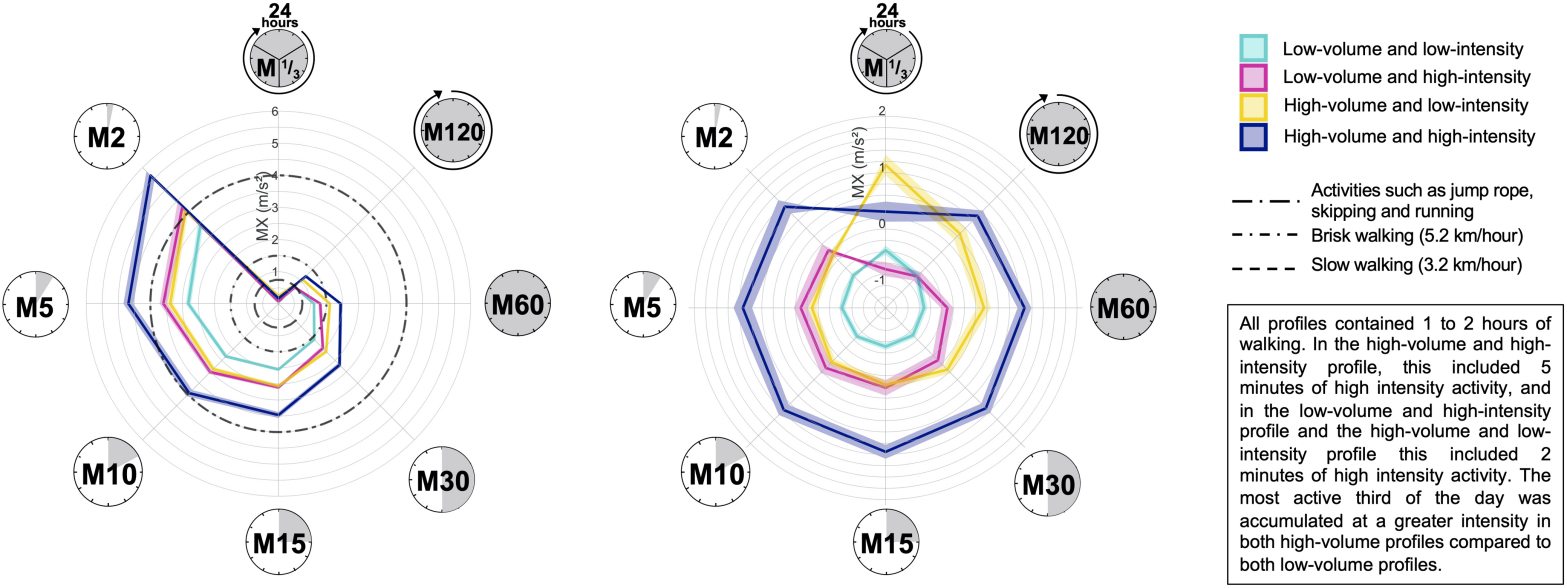
Illustration of the physical activity profile for raw (left) and standardized (right) MX metrics in males. Profiles are presented for four groups, (1) high‐volume and high‐intensity, (2) high‐volume and low‐intensity, (3) low‐volume and high‐intensity, and (4) low‐volume and low‐intensity. High‐volume was defined as average‐acceleration ≥ mean, low‐volume was defined as average‐acceleration < mean, high‐intensity was defined as intensity‐gradient ≥ mean, low‐intensity was defined as intensity‐gradient < mean. Values are the mean; error ribbons are the standard error of the mean. High‐volume was defined as average‐acceleration ≥ mean, low‐volume was defined as average‐acceleration < mean, high‐intensity was defined as intensity‐gradient ≥ mean, low‐intensity was defined as intensity‐gradient < mean. The MX metrics show the acceleration above which a child's most active X minutes are accumulated. Each plot shows (clockwise) *M1/3*, the intensity above which a child's most active 480 min are accumulated; *M120*, the intensity above which a child's most active 120 min are accumulated; *M60*, the intensity above which a child's most active 60 min are accumulated; *M30*, the intensity above which a child's most active 30 min are accumulated; *M15*, the intensity above which a child's most active 15 min are accumulated; *M10*, the intensity above which a child's most active 10 min are accumulated; *M5*, the intensity above which a child's most active 5 min are accumulated; *M2*, the intensity above which a child's most active 2 min are accumulated.

The raw and standardized MX metrics for low and high volume and intensity profiles are presented in Figure 4 for females and in Figure 5 for males. On average, all profiles accumulated 60 min at an intensity equivalent to slow to brisk walking. In the high‐volume and high‐intensity group this included 5 min of high‐intensity activity, and in the high‐volume and low‐intensity and the low‐volume and high‐intensity groups this included 2 min of high intensity activity. The high‐volume groups, with either low‐intensity or high‐intensity, accumulated more light activity compared to the low‐volume groups, with 2 h at an intensity equivalent to slow walking in females and males. The most active third of the day was accumulated at a high intensity by the high‐volume groups compared to the low‐volume groups in females and males.

## 4 DISCUSSION

This study is the first to examine the associations of average‐acceleration, as a measure of PA volume, and intensity‐gradient, as a measure of PA intensity distribution, with TBLH BMC, lean mass, and fat mass deriving from DXA in a population sample of pre‐ and early‐pubertal children, alongside assessing the associations between MVPA and these outcomes. Greater PA volume was associated with increased BMC in females, and with increased BMC and lean mass, and reduced fat mass, in males. A higher intensity distribution was associated with reduced BMC in males. Although the novel approach revealed unique relationships for bone outcomes, MVPA was positively associated with lean mass and negatively associated with fat mass in females and males and was potentially better than our new approach at capturing activity relevant to lean and fat mass in females. Even so, it remains unclear whether it was volume or intensity of MVPA driving these associations, highlighting the utility of independent metrics which capture both the volume and intensity of PA. Adjusting for lean and fat mass altered the relationships between PA with BMC, emphasizing the importance of considering lean and fat mass alongside bone. The high‐volume PA profiles included short periods of high intensity activity, with several hours of light activity, suggesting that increasing PA at any intensity may improve BMC and lean mass and reduce fat mass in children.

Compared to reference values for children aged 9 years, the children in our study had typical median levels of TBLH aBMD and lean mass,^28^, ^29^, ^30^ though our study had a lower proportion of children living with obesity than the International Study of Childhood Obesity, Lifestyle and the Environment (ISCOLE).^31^ It is difficult to compare PA levels between study samples as the amount of PA reported is dependent on accelerometer protocol and placement, epoch lengths, wear time algorithms, and intensity cut‐points.^32^ Even so, the children in our sample may be more active than children aged 9–11 years in ISCOLE study.^31^

### 4.1 Bone mineral content

We did not observe an association between MVPA and TBLH BMC, adjusting for lean and fat mass, which highlights the importance of considering PA exposures relating to mechanical loading rather than energy expenditure when examining the relationship between PA and bone mass. Our findings indicated that a greater volume of PA is associated with favorable bone outcomes in pre‐ and early‐pubertal children, and this association is partially explained by lean mass. However, for a given volume of PA, males accumulating the volume at a lower intensity had greater TBLH BMC, though this was likely driven by the association at the upper‐limb only. The inverse relationship between intensity distribution and BMC in males we observed is unexpected, given that bone responds positively to strains brought about by high impact loading cycles.^3^ This observation may be because the types of osteogenic activities that males were engaging in were not captured in the high end of the intensity spectrum.^3^ In children ages 6–11 years, accelerometer counts during jumping, captured in 15 s epochs, were lower than those observed during running, despite ground reaction forces being greater.^20^ This disassociation between accelerometer counts and ground reaction forces during jumping may be further magnified with longer epoch lengths, and therefore, the use of the 60‐s epoch length may not have captured high impact activity. This may have been more apparent in males, due to potential sex and gender differences in engagement in PA types, with girls in Finland participating in less high intensity activity than boys, which is likely to result in differences in osteogenic responses.^33^ Therefore, the types of activities which are positively associated with BMC may not have been reflected within the intensity‐gradient, and sex and gender differences in activity participation may explain why the inverse relationship between intensity with BMC was only observed in males.

The Iowa Bone Development Study (IBDS) found that in participants age 17–23 years, both average‐acceleration and intensity‐gradient were positively associated with TBLH BMC, after adjustment for similar covariates to those used in our analysis.^7^ Although lean and fat mass were not separated as covariates, total mass was adjusted for, meaning our final models are comparable.^7^ The IBDS used a 5‐s epoch to calculate the intensity‐gradient, so may have been more likely to capture osteogenic activity, and therefore more likely to find positive associations between intensity‐gradient with bone outcomes. In addition, PA levels may account for the different findings, as we observed higher levels of mean daily MVPA compared to the IBDS sample, and our MVPA levels are likely an underestimation due to the 60‐s epoch used to capture the accelerometer data, compared to the 5‐s epoch used in the IBDS.^34^ Further, our participants were younger than those from the IBDS sample, which may contribute to the different observations, as maturation may moderate the skeletal response to mechanical loading.^5^ Therefore, the differences in maturity status and PA levels may account for the different findings between our study and the IBDS.^7^

In our study and the IBDS, the high‐volume activity profiles were characterized by relatively low amounts of high‐intensity activity, at least 60 min of MVPA, and several hours of being lightly active.^7^ Our findings indicate that for active children, greater PA volume may elicit positive bone outcomes, and accruing several hours of light‐intensity, weight‐bearing activity may also be beneficial for BMC.

### 4.2 Lean mass

The positive association between average‐acceleration and lean mass was evident in males only when controlling for intensity‐gradient. However, the association in females was approaching statistical significance, so is likely due to reduced statistical power, lower lean mass and lower average‐acceleration in females compared to males. Further, MVPA was positively associated with lean mass in females and males. Previous studies have not examined average‐acceleration and intensity‐gradient in relation to lean mass, though research has found that more active children and adolescents have greater lean mass, similar to the positive associations we observed between MVPA with lean mass.^2^, ^35^ However, these studies have been unable to separate the associations of PA volume and intensity, as all summary intensities of PA should not be included in the same model due to issues with multicollinearity.^2^, ^35^ This further highlights the potential benefits of using the accelerometer metrics proposed by Rowlands and colleagues,^9^ as it allows the full intensity spectrum to be characterized and analyzed without issues of multicollinearity, though other methods have been proposed which also account for this.^36^ Our findings indicate that PA volume and MVPA are important for lean mass.

### 4.3 Fat mass

Of the previous studies that have examined the associations between average‐acceleration and intensity‐gradient with measures of adiposity in children, ours is the only one to observe an independent relationship between average‐acceleration and a measure of adiposity, albeit only in males. It is unclear what accounts for the null finding we observed in females. Levels of fat mass and the prevalence of overweight and obesity did not differ between females and males, though the females were less active in terms of PA volume and MVPA than the males. It is therefore possible that the females were not accumulating enough weight‐bearing PA to observe an association with fat mass, as we did observe a negative association between MVPA with fat mass in females and males.

Intensity‐gradient was independently negatively associated with BIA‐assessed body fat percentage in adolescent girls,^9^ and with waist‐to‐height ratio in primary school children.^37^ Furthermore, multivariate pattern analysis demonstrated that PA at vigorous intensities had the strongest relationship with waist‐to‐height ratio in children ages 5–18 years, emphasizing the importance of accruing PA at the high end of the intensity spectrum.^38^ Although our sample had a lower prevalence of children living overweight and obesity compared to these previous studies, differences in epoch length, which are discussed below, likely account for the differences in findings.^9^, ^37^, ^38^ Our findings suggest that greater PA volume is important for reducing fat mass in males in this cohort, while MVPA may reduce fat mass in females and males in this cohort, with a relatively low prevalence of children living with overweight and obese weight status.

### 4.4 Considerations

The accelerometry methodology may explain the differences between our findings and those which have found PA intensity to be equally or more important than volume for both BMC and fat mass.^7^, ^9^, ^37^ In our study, accelerometer data was averaged over 60‐s, whereas data in more recent studies examining intensity‐gradient has been averaged over 5‐s.^7^, ^9^, ^37^ As high intensity activity is accrued in short bursts in children (typically in bouts <10‐s), it is impossible to capture these short bursts of high‐intensity activity with a 60‐s epoch.^34^ As such, our intensity‐gradient metric may be blunted, limiting our ability to detect an effect. This is supported by multivariate pattern analysis of the associations between PA intensity signature of varying epoch lengths with metabolic health, which has shown that the intensity most strongly associated with metabolic health is lower the longer the epoch, reflecting the dilution over longer epoch lengths.^39^ Given that many large longitudinal studies,^1^, ^2^ as well as the International Children's Accelerometry Database,^38^ have accelerometer data in 60‐s epochs, investigating the effect of epoch length on intensity‐gradient should be a priority before this metric is applied in research using longer epochs. Further, given the high levels of PA and low levels of fat mass in our sample, it is unknown whether these findings extend to less active children and to children living with overweight and obesity. Although we adjusted for important covariates, residual confounding remains a potential limitation in all observational studies, and causality cannot be assumed as bidirectional relationships are possible.

The strengths of our study include the population‐based sample of children, device‐measured PA, the comparison of novel accelerometer metrics with MVPA estimates based on energy expenditure, and the measurement of BMC, lean mass, and fat mass by DXA. The application of novel accelerometer metrics to characterize volume and intensity allowed the independent contributions of each of these aspects of PA to be examined. The translation of the findings with MX metrics makes the findings easy to understand and apply to TBLH BMC, lean mass, and fat mass, and we have been able to provide clinically relevant recommendations.

### 4.5 Clinical relevance

A 1 SD greater average‐acceleration was independently associated with 0.02 kg greater TBLH BMC in females, and with 0.03 kg greater TBLH BMC and 0.51 kg greater TBLH lean mass in males. This equates to ~2% of the annual growth in TBLH BMC in females and males, and ~18% of the annual growth in TBLH lean mass in males.^28^, ^40^ Given that ~40% of adult total body BMC is accrued in the 4 years surrounding peak height velocity, accruing more bone during this period is important for increasing peak bone mass in young adulthood.^41^ A 1 SD greater average‐acceleration was associated with 1.4 kg lower TBLH fat mass in males, which equates to ~4% lower body fat percentage. It is therefore likely that our findings are clinically meaningful.

In females, a 1 SD (0.0579 m/s^2^) increase in average‐acceleration could be achieved by replacing time per day spent at the average‐acceleration level with an accumulation of:

1. 22 min of high‐intensity activities such as running, jumping, and skipping OR
2. 64 min of brisk walking OR
3. 2.5 h of slow walking/light activity OR
4. any combination of the above such that the sum of the increases is equal to 0.0579 m/s^2^, as described in Materials and methods.

In males, a 1 SD (0.0696 m/s^2^) increase in average‐acceleration could be achieved by replacing time per day spent at the average‐acceleration level with:

1. 27 min of high‐intensity activities such as running, jumping, and skipping OR
2. 78 min of brisk walking OR
3. 3 h of slow walking/light activity OR
4. any combination of the above such that the sum of the increases is equal to 0.0705 m/s^2^, as described in Materials and methods.

This demonstrates the practical applications of our findings.

## 5 CONCLUSION

A higher volume of PA was associated with greater BMC in females, and with greater BMC and lean mass, and reduced fat mass in males, while more MVPA was associated with greater lean mass and lower fat mass in females and males, in a population sample of children aged 9–11 years. The relationship between PA volume and BMC was influenced by lean and fat mass, and body composition should be considered in future research to better understand the association between PA and BMC. The high‐volume PA profiles were characterized by at least 2 min of high‐intensity activity, as well as several hours of light activity. These findings support the current World Health Organization recommendations to increase total activity, though our analyses need replicating with accelerometer data averaged over shorter epochs.^21^ These intricacies would have been missed if only MVPA was explored as the exposure of interest. This highlights the importance of using methods that account for the whole PA profile and which align PA with mechanical loading rather than energy expenditure when considering bone as an outcome.

### 5.1 Perspectives

In the current study, we investigated the associations between PA volume, PA intensity, and MVPA with BMC, lean mass and fat mass in children aged 9–11 years. We found that high PA volume was important for improved BMC in females, and for improved BMC and lean mass, and reduced fat mass in males, while high levels of MVPA were important for greater lean mass and lower fat mass in females and males, in a population sample of children aged 9–11 years. Our results confirm findings from previous studies indicating the importance of PA for bone, lean and fat mass in children, and extend the current literature by considering both the volume and the full spectrum of PA intensity alongside MVPA, highlighting importance of using methods that account for whole PA profile. Further, we translated our findings to provide a meaningful description of the PA profile associated with more favorable BMC, lean and fat mass, which included short periods of high intensity activity as well as several hours of light activity.

## FUNDING INFORMATION

This work was financially supported by grants from the Ministry of Education and Culture of Finland, Ministry of Social Affairs and Health of Finland, Finnish Innovation Fund Sitra, Social Insurance Institution of Finland, Finnish Cultural Foundation, Juho Vainio Foundation, Foundation for Pediatric Research, Doctoral Programs in Public Health, Paavo Nurmi Foundation, Paulo Foundation, Diabetes Research Foundation, The Finnish Medical Society Duodecim, Orion Research Foundation sr, Research Committee of the Kuopio University Hospital Catchment Area (State Research Funding), Kuopio University Hospital (previous state research funding [EVO], funding number 5031343) and the city of Kuopio. SB and KW were supported by the UK Medical Research Council (MC_UU_12015/3 and MC_UU_00006/4) and the NIHR Cambridge Biomedical Research Centre (IS‐BRC‐1215‐20 014). AR is supported by the National Institute for Health Research (NIHR) Leicester Biomedical Research Centre, the NIHR Applied Research Collaborations—East Midlands. The funders had no role in the design and conduct of the study; collection, management, analysis, and interpretation of the data; preparation, review, or approval of the manuscript; and decision to submit the manuscript for publication.

## CONFLICT OF INTEREST

The authors declare that they have no competing interests.

## Supporting information


Appendix S1

Appendix S2

Appendix S3

## Data Availability

The datasets analyzed during the current study are not publicly available due to research ethical reasons and because the owner of the data is the University of Eastern Finland and not the research group. Requests to access the datasets should be directed to www.panicstudy.fi/en/etusivu.

## Footnotes

* When referring to the sex or gender of participants in previous studies, we have used the terminology presented in the previous studies.
