# Supplementary material for: Physical activity volume and intensity distribution in relation to bone, lean and fat mass in children
Source: Scand J Med Sci Sports. 2022 Nov 17;33(3):267–82. doi: 10.1111/sms.14255 (PMC10947490; doi:10.1111/sms.14255)
Supplement: Supplementary file 1 — Appendix S1 [file SMS-33-267-s002.pdf]

Additional File 1. Supplementary Tables

Table S1. Differences in characteristics of children included and children excluded from analyses

	**Included children (n = 290)**	**Excluded children (n = 127 to 147)**	***p* value for difference**
**Age (years)**	9.8 (0.4)	9.7 (0.4)	0.28
**Stature (cm)**	140.8 (6.1)	139.8 (6.6)	0.12
**Weight (kg)**	32.9 (29.5 to 38.7)	33.2 (28.9 to 38.7)	0.84
**Pubertal Status (n, %)**			
**prepubertal**	218 (75.1)	104 (80.0)	0.28
**pubertal**	72 (24.9)	26 (20.0)	
**IOTF Definition (n, %)**			
**thin**	31 (10.7)	13 (8.9)	0.09
**normal weight**	216 (74.5)	100 (68.0)	
**overweight**	33 (11.4)	30 (20.4)	
**obese**	10 (3.4)	4 (2.7)	
**TBLH BMC (kg)**	0.9 (0.2)	0.9 (0.2)	0.46
**TBLH lean mass (kg)**	21.8 (2.9)	21.8 (3.1)	0.90
**TBLH fat mass (kg)**	6.7 (4.4 to 10.3)	7.2 (4.0 to 12.0)	0.49

The values are means (standard deviations), medians (interquartile ranges) or numbers (percentages) of children, and *p*-values are for the differences between females and males. Differences between included and excluded children were tested with independent samples *t* test for continuous variables with normal distributions, with Mann-Whitney U test for continuous variables with skewed distributions, and with Fishers exact test for categorical variables.
Number of included and excluded children: 290 and 147 for age, stature, body weight, and IOTF weight status; 290 and 130 for pubertal status; 290 and 127 for TBLH BMC, TBLH lean mass, and TBLH fat mass
BMI-SDS, Body mass index standard deviation score; *IOTF,* International Obesity Task Force; *TBLH,* Total body less head; *BMC*, bone mineral content.

Table S2. Associations of physical activity volume (average-acceleration) and intensity distribution (intensity-gradient) with lower-limb and upper-limb bone mineral content, lean mass, and fat mass in 158 females.

	**Model 1 (adjusted for age, stature, pubertal status, and wear time)**		**Model 2 (Model 1 + alternate activity metric)**		**Model 3 (Model 2 + Intensity X Volume interaction)**		**Model 4 (Model 3 + limb-specific lean mass)**		**Model 5 (Model 3 + limb-specific fat mass)**		**Model 6 (Model 3 + limb-specific lean mass and fat mass)**	
	ß (95% CI)	*p*	ß (95% CI)	*p*	ß (95% CI)	*p*	ß (95% CI)	*p*	ß (95% CI)	*p*	ß (95% CI)	*p*
**Lower-limb BMC**												
Intensity[a]	46.052 (-12.802 to 104.905)	0.124	15.354 (-57.709 to 88.416)	0.679	18.929 (-55.258 to 93.115)	0.615	25.829 (-37.916 to 89.574)	0.425	23.684 (-31.224 to 78.593)	0.395	28.480 (-18.166 to 75.127)	0.230
Volume[b]	**148.325 (5.268 to 291.383)**	**0.042**	125.957 (-52.674 to 304.589)	0.166	137.616 (-45.576 to 320.807)	0.140	49.130 (-109.991 to 208.250)	0.543	**158.560 (22.937 to 294.183)**	**0.022**	87.583 (-29.041 to 204.207)	0.140
Intensity X Volume					-295.433 (-1,279.780 to 688.914)	0.554	-661.329 (-1,512.454 to 189.796)	0.127	-155.748 (-884.640 to 573.145)	0.673	-455.382 (-1,079.197 to 168.433)	0.151
**Upper-limb BMC**												
Intensity[a]	9.286 (-6.958 to 25.531)	0.260	1.391 (-18.792 to 21.575)	0.892	2.514 (-17.973 to 23.001)	0.809	9.808 (-5.677 to 25.292)	0.213	4.209 (-14.806 to 23.224)	0.662	10.403 (-4.201 to 25.008)	0.161
Volume[b]	34.421 (-5.078 to 73.921)	0.087	32.394 (-16.952 to 81.741)	0.197	36.055 (-14.534 to 86.645)	0.161	3.820 (-34.732 to 42.372)	0.845	37.090 (-9.836 to 84.017)	0.120	7.037 (-29.345 to 43.420)	0.703
Intensity X Volume					-92.768 (-364.601 to 179.064)	0.501	-194.592 (-400.136 to 10.952)	0.063	-67.590 (-319.925 to 184.746)	0.597	-169.401 (-363.555 to 24.752)	0.087
**Lower-limb lean mass**												
Intensity[a]	536.556 (-196.191 to 1,269.303)	0.150	-58.680 (-959.962 to 842.602)	0.898	-163.741 (-1,073.855 to 746.373)	0.723	.	.	-146.893 (-1,042.679 to 748.893)	0.746	.	.
Volume[b]	**2,356.843 (593.029 to 4,120.657)**	**0.009**	**2,442.331 (238.772 to 4,645.889)**	**0.030**	2,099.707 (-147.682 to 4,347.095)	0.067	.	.	2,173.905 (-38.669 to 4,386.479)	0.054	.	.
Intensity X Volume					8,682.442 (-3,393.505 to 20,758.389)	0.157	.	.	9,177.293 (-2,713.979 to 21,068.565)	0.129	.	.
**Upper-limb lean mass**												
Intensity[a]	65.731 (-191.948 to 323.411)	0.615	-143.638 (-460.583 to 173.306)	0.372	-172.835 (-493.693 to 148.023)	0.289	.	.	-159.155 (-475.021 to 156.711)	0.321	.	.
Volume[b]	**649.813 (27.940 to 1,271.686)**	**0.041**	**859.071 (84.168 to 1,633.975)**	**0.030**	763.857 (-28.454 to 1,556.167)	0.059	.	.	772.210 (-7.325 to 1,551.745)	0.052	.	.
Intensity X Volume					2,412.845 (-1,844.496 to 6,670.186)	0.265	.	.	2,616.037 (-1,575.714 to 6,807.788)	0.219	.	.
**Lower-limb fat mass**												
Intensity[a]	-582.718 (-2,367.452 to 1,202.015)	0.520	-291.318 (-2,519.748 to 1,937.112)	0.797	-214.931 (-2,478.985 to 2,049.124)	0.851	-135.499 (-2,364.588 to 2,093.590)	0.905	.	.	.	.
Volume[b]	-1,620.059 (-5,981.833 to 2,741.716)	0.464	-1,195.654 (-6,643.979 to 4,252.671)	0.665	-946.541 (-6,537.281 to 4,644.199)	0.738	-1,965.119 (-7,529.352 to 3,599.113)	0.486	.	.	.	.
Intensity X Volume					-6,312.780 (-36,353.640 to 23,728.080)	0.679	-10,524.677 (-40,287.374 to 19,238.020)	0.486	.	.	.	.
**Upper-limb fat mass**												
Intensity[a]	-181.354 (-629.054 to 266.346)	0.425	-145.406 (-704.670 to 413.859)	0.608	-123.253 (-691.349 to 444.842)	0.669	-63.117 (-624.138 to 497.905)	0.824	.	.	.	.
Volume[b]	-359.334 (-1,454.714 to 736.045)	0.518	-147.501 (-1,514.856 to 1,219.854)	0.832	-75.259 (-1,478.085 to 1,327.568)	0.916	-341.038 (-1,737.788 to 1,055.712)	0.630	.	.	.	.
Intensity X Volume					-1,830.692 (-9,368.533 to 5,707.148)	0.632	-2,670.226 (-10,117.183 to 4,776.730)	0.480	.	.	.	.

The values are unstandardised regression coefficients (ß), 95% confidence intervals (CI), and *p* values from linear regression models. Model 1 included the activity variable (average-acceleration or intensity-gradient) adjusted for age, stature, pubertal status, and accelerometer wear time, Model 2 included additional adjustment for the alternate activity metric (average-acceleration or intensity-gradient), Model 3 additionally included the interaction term for average-acceleration by intensity-gradient. For BMC as the outcome, Model 4 included additional adjustment for lean mass, Model 5 included additional adjustment for fat mass, and Model 6 included adjustment for lean and fat mass. For lean mass as the outcome, the final model (Model 5) included adjustment for fat mass, and for fat mass at the outcome, the final model (Model 4) included adjustment for lean mass.
Bold emphasis indicates statistical significance at $p < 0.05$.

[a]Intensity is reflected in the intensity-gradient, calculated from data collected with a 24-hour protocol adjusted for diurnal imbalance in non-wear, as the regression line from log-log plot of intensity (x) and fraction of wear time accumulated (y).
[b]Volume is reflected in the average-acceleration across data, collected with a 24-hour protocol adjusted for diurnal imbalance in non-wear.
Activity variables were mean-centred before entry into analysis, with interaction terms computed from the centred scores.

BMC, bone mineral content.

Table S3. Associations of physical activity volume (average-acceleration) and intensity distribution (intensity-gradient) with lower-limb and upper-limb bone mineral content, lean mass, and fat mass in 112 males.

	**Model 1 (adjusted for age, stature, pubertal status, and wear time)**		**Model 2 (Model 1 + alternate activity metric)**		**Model 3 (Model 2 + Intensity X Volume interaction)**		**Model 4 (Model 3 + limb-specific lean mass)**		**Model 5 (Model 3 + limb-specific fat mass)**		**Model 6 (Model 3 + limb-specific lean mass and fat mass)**	
	ß (95% CI)	*p*	ß (95% CI)	*p*	ß (95% CI)	*p*	ß (95% CI)	*p*	ß (95% CI)	*p*	ß (95% CI)	*p*
**Lower-limb BMC**												
Intensity[a]	-6.010 (-67.683 to 55.662)	0.847	-45.045 (-130.657 to 40.566)	0.300	-47.195 (-132.645 to 38.255)	0.276	-58.342 (-127.263 to 10.579)	0.096	-29.400 (-88.947 to 30.148)	0.330	-40.262 (-89.720 to 9.195)	0.110
Volume[b]	64.901 (-96.174 to 225.976)	0.427	146.924 (-77.205 to 371.054)	0.197	108.059 (-123.228 to 339.347)	0.357	38.808 (-148.341 to 225.957)	0.682	**285.590 (121.767 to 449.413)**	**0.001**	**206.170 (68.744 to 343.596)**	**0.004**
Intensity X Volume					444.936 (-234.608 to 1,124.480)	0.197	317.243 (-231.301 to 865.788)	0.255	-17.883 (-497.437 to 461.671)	0.941	-23.494 (-421.129 to 374.141)	0.907
**Upper-limb BMC**												
Intensity[a]	-15.001 (-30.813 to 0.812)	0.063	**-32.703 (-54.336 to -11.069)**	**0.003**	**-33.132 (-54.780 to -11.485)**	**0.003**	-11.868 (-27.338 to 3.601)	0.131	**-31.278 (-49.498 to -13.057)**	**0.001**	**-14.347 (-28.457 to -0.238)**	**0.046**
Volume[b]	7.079 (-34.874 to 49.033)	0.739	**66.628 (9.991 to 123.265)**	**0.022**	**58.861 (0.268 to 117.454)**	**0.049**	5.325 (-36.390 to 47.041)	0.801	**92.858 (42.688 to 143.029)**	**0.000**	33.980 (-5.545 to 73.505)	0.091
Intensity X Volume					88.919 (-83.234 to 261.072)	0.309	86.303 (-33.276 to 205.883)	0.156	-0.949 (-147.869 to 145.972)	0.990	34.648 (-75.964 to 145.259)	0.536
**Lower-limb lean mass**												
Intensity[a]	632.376 (-65.539 to 1,330.291)	0.075	224.579 (-745.208 to 1,194.366)	0.648	212.803 (-760.159 to 1,185.764)	0.666	.	.	300.559 (-628.816 to 1,229.934)	0.523	.	.
Volume[b]	**1,943.862 (125.583 to 3,762.140)**	**0.036**	1,534.921 (-1,003.961 to 4,073.803)	0.234	1,321.995 (-1,311.523 to 3,955.513)	0.322	.	.	2,197.483 (-359.349 to 4,754.315)	0.091	.	.
Intensity X Volume					2,437.628 (-5,299.903 to 10,175.159)	0.534	.	.	155.247 (-7,329.294 to 7,639.788)	0.967	.	.
**Upper-limb lean mass**												
Intensity[a]	-149.902 (-395.872 to 96.067)	0.230	**-457.228 (-791.949 to -122.506)**	**0.008**	**-457.500 (-793.841 to -121.159)**	**0.008**	.	.	**-437.362 (-749.677 to -125.048)**	**0.006**	.	.
Volume[b]	324.179 (-320.944 to 969.302)	0.322	**1,156.753 (280.460 to 2,033.047)**	**0.010**	**1,151.838 (241.463 to 2,062.213)**	**0.014**	.	.	**1,521.000 (661.035 to 2,380.965)**	**0.001**	.	.
Intensity X Volume					56.273 (-2,618.497 to 2,731.043)	0.967	.	.	-919.561 (-3,437.910 to 1,598.788)	0.471	.	.
**Lower-limb fat mass**												
Intensity[a]	**-2,042.183 (-3,808.822 to -275.543)**	**0.024**	-611.230 (-3,053.071 to 1,830.611)	0.621	-699.067 (-3,118.855 to 1,720.720)	0.568	-864.301 (-3,174.390 to 1,445.788)	0.460	.	.	.	.
Volume[b]	**-6,499.014 (-11,077.933 to -1,920.096)**	**0.006**	-5,386.016 (-11,778.705 to 1,006.674)	0.098	**-6,974.167 (-13,523.815 to -424.520)**	**0.037**	**-8,000.648 (-14,273.475 to -1,727.821)**	**0.013**	.	.	.	.
Intensity X Volume					18,181.533 (-1,061.964 to 37,425.030)	0.064	16,288.805 (-2,097.196 to 34,674.805)	0.082	.	.	.	.
**Upper-limb fat mass**												
Intensity[a]	-454.477 (-923.142 to 14.187)	0.057	-77.149 (-725.022 to 570.724)	0.814	-100.732 (-742.537 to 541.073)	0.757	232.294 (-380.596 to 845.184)	0.455	.	.	.	.
Volume[b]	**-1,560.722 (-2,774.686 to -346.757)**	**0.012**	-1,420.239 (-3,116.357 to 275.879)	0.100	**-1,846.620 (-3,583.796 to -109.444)**	**0.037**	**-2,685.072 (-4,337.798 to -1,032.345)**	**0.002**	.	.	.	.
Intensity X Volume					4,881.308 (-222.683 to 9,985.298)	0.061	**4,840.345 (102.765 to 9,577.925)**	**0.045**	.	.	.	.

The values are regression coefficients (ß), 95% confidence intervals (CI), and *p* values from linear regression models. Model 1 included the activity variable (average-acceleration or intensity-gradient) adjusted for age, stature, pubertal status, and accelerometer wear time, Model 2 included additional adjustment for the alternate activity metric (average-acceleration or intensity-

gradient), Model 3 additionally included the interaction term for average-acceleration by intensity-gradient. For BMC as the outcome, Model 4 included additional adjustment for lean mass, Model 5 included additional adjustment for fat mass, and Model 6 included adjustment for lean and fat mass. For lean mass as the outcome, the final model (Model 5) included adjustment for fat mass, and for fat mass at the outcome, the final model (Model 4) included adjustment for lean mass.
Bold emphasis indicates statistical significance at $p < 0.05$.
[a]Intensity is reflected in the intensity-gradient, calculated from data collected with a 24-hour protocol adjusted for diurnal imbalance in non-wear, as the regression line from log-log plot of intensity (x) and fraction of wear time accumulated (y).
[b]Volume is reflected in the average-acceleration across data, collected with a 24-hour protocol adjusted for diurnal imbalance in non-wear.
Activity variables were mean-centred before entry into analysis, with interaction terms computed from the centred scores.

BMC, bone mineral content.

Table S4. Associations of moderate-to-vigorous physical activity with total body less head bone mineral content, lean mass, and fat mass (n = 288, 157 females and 131 males).

	**Model 1 (adjusted for age, sex, stature, and pubertal status)**		**Model 2 (Model 1 + lean mass)**		**Model 3 (Model 1 + fat mass)**		**Model 4 (Model 1 + lean mass and fat mass)**	
	ß (95% CI)	*p*	ß (95% CI)	*p*	ß (95% CI)	*p*	ß (95% CI)	*p*
**TBLH BMC**								
Females	-0.000 (-0.001 to 0.000)	0.194	**0.001 (0.000 to 0.001)**	**0.001**	0.000 (-0.000 to 0.001)	0.137	0.000 (-0.000 to 0.001)	0.545
Males	-0.000 (-0.000 to 0.000)	0.647	-0.000 (-0.001 to 0.000)	0.084	**0.000 (0.000 to 0.001)**	**0.005**	0.000 (-0.000 to 0.000)	0.120
**TBLH lean mass**								
Females	**0.010 (0.004 to 0.015)**	**0.001**	**.**	**.**	**0.012 (0.006 to 0.018)**	**0.000**	.	.
Males	0.004 (-0.000 to 0.009)	0.066	.	.	**0.007 (0.002 to 0.012)**	**0.006**	.	.
**TBLH fat mass**								
Females	**-0.023 (-0.039 to -0.008)**	**0.003**	**-0.030 (-0.045 to -0.014)**	**0.000**	.	.	.	.
Males	**-0.026 (-0.038 to -0.014)**	**0.000**	**-0.029 (-0.041 to -0.017)**	**0.000**	.	.	.	.

The values are regression coefficients (ß), 95% confidence intervals (CI), and *p* values from linear regression models. Model 1 included moderate-to-vigorous physical activity adjusted for age, stature, pubertal status, and accelerometer wear time. For bone mineral content as the outcome, Model 2 included additional adjustment for lean mass, Model 3 included additional adjustment for fat mass, and Model 4 included adjustment for lean and fat mass. For lean mass as the outcome, the final model (Model 3) included adjustment for fat mass, and for fat mass at the outcome, the final model (Model 2) included adjustment for lean mass.
Bold emphasis indicates statistical significance at $p < 0.05$.
TBLH, Total body less head; *BMC*, bone mineral content.
