# Supplementary material for: Physical activity volume and intensity distribution in relation to bone, lean and fat mass in children
Source: Scand J Med Sci Sports. 2022 Nov 17;33(3):267–82. doi: 10.1111/sms.14255 (PMC10947490; doi:10.1111/sms.14255)
Supplement: Supplementary file 2 — Appendix S2 [file SMS-33-267-s003.pdf]

Additional File 2. Methods for calculating intensity-gradient metric

To calculate the intensity-gradient, the acceleration signal was summarised as a fraction of wear time spent in 25 acceleration thresholds (m/s^2) (0.075, 0.25, 0.5, 0.75, 1, 1.25, 1.5, 1.75, 2, 2.25, 2.5, 2.75, 3, 3.25, 3.5, 3.75, 4, 4.25, 4.5, 4.75, 5, 6, 7, 8, 9) across the movement intensity continuum. These thresholds are used to provide greater resolution at the low end of the intensity spectrum, where the data are denser, and less resolution at the high end of the intensity spectrum, where the data are sparser. To generate the variable, the natural log of intensity (the midpoint of each intensity bin) and the fraction of wear time (adjusted for diurnal imbalance in non-wear) accumulated in each intensity bin was calculated. These variables were entered into a regression model, with the natural log of fraction of time accumulated as the outcome and the natural log of the midpoint of the intensity bin as the predictor. The regression equation is as follows:

$$\log(\textit{fraction of wear time accumulated at each intensity}) = b_0 + b_1(\log(\textit{midpoint of each intensity bin}))$$

The process for calculating the intensity-gradient is shown in Additional File 2, Figure S1.

The mean R^2 of the log-log regression, reflective of the fit of the regression line, was 0.89 (SD = 0.03, range = 0.77 to 0.96). The cases with a lower R^2, around 0.80*,* indicating a poorer linear fit, tended to have relatively more very light intensity activity, and relatively less sedentary time and high intensity activity compared to cases with a higher R^2*.* In these cases, the intensity-gradient was mid-range in the sample, indicating that although the fit was worse, the intensity-gradient was still capturing the distribution of intensity across the spectrum.

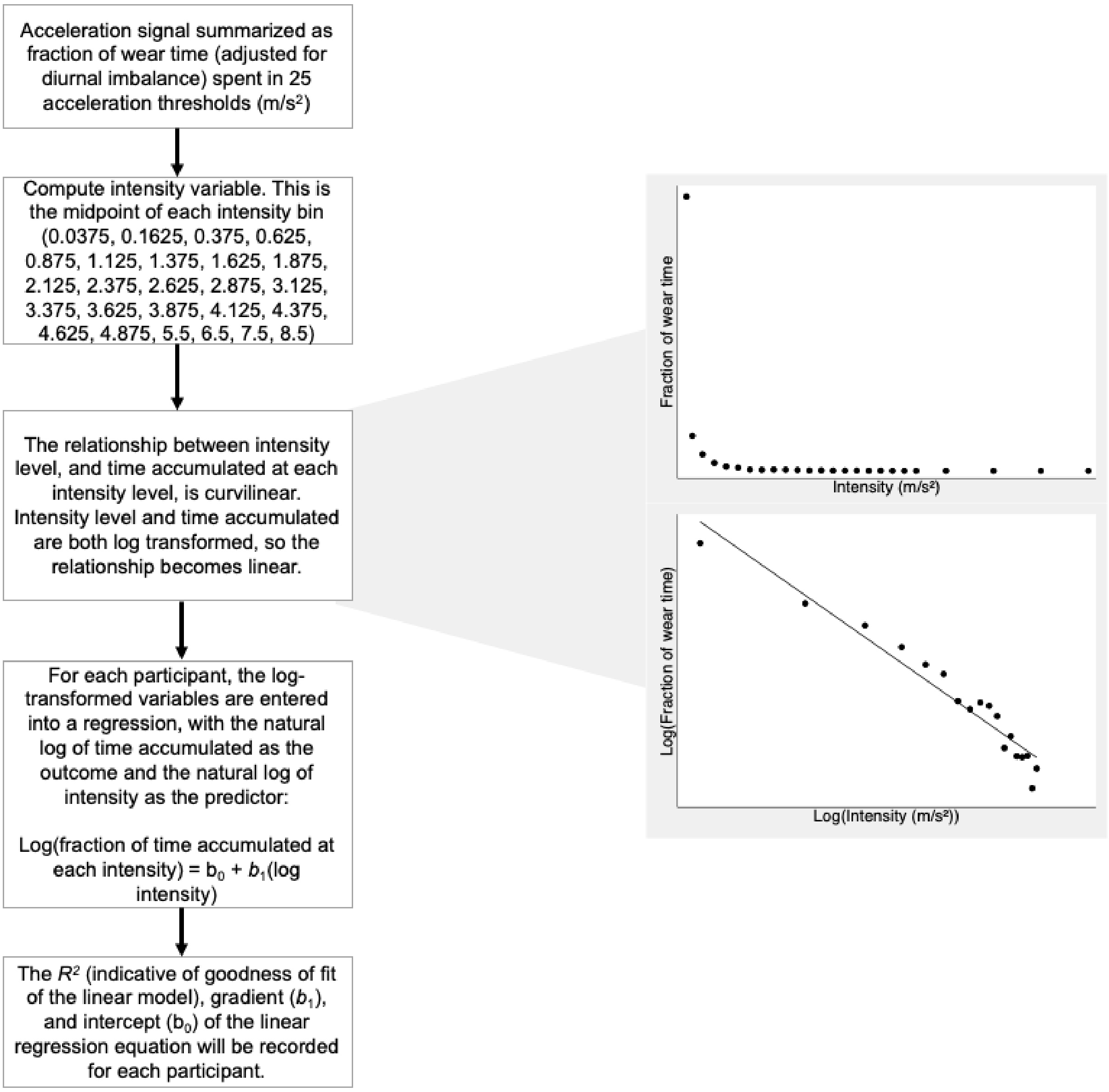

Figure S1. Flow chart demonstrating the process of calculating the intensity gradient.

Example plots are from the raw and log-transformed intensity distribution from one participant, with the fitted linear regression line for the log-transformed data.
