# Supplementary material for: Physical activity volume and intensity distribution in relation to bone, lean and fat mass in children
Source: Scand J Med Sci Sports. 2022 Nov 17;33(3):267–82. doi: 10.1111/sms.14255 (PMC10947490; doi:10.1111/sms.14255)
Supplement: Supplementary file 3 — Appendix S3 [file SMS-33-267-s001.pdf]

Additional File 3. The accumulation of physical activity to increase average-acceleration by 1 standard deviation

To demonstrate how physical activity could be accumulated for a 1 standard deviation greater average-acceleration, we applied the calculation by Rowlands and colleagues, assuming that the introduced activity would replace time spent at the mean average-acceleration: 1440 x (standard deviation of average-acceleration)/(acceleration associated with a specific activity – mean average-acceleration) (1). In our analysis, the SD of average-acceleration was 0.0579 m/s^2 in females, and 0.0696 m/s^2 in males.

We used the following calculation to demonstrate how this increase could be achieved through high-intensity activities equivalent to 4 m/s^2 in females:
1440 x (0.0579)/(4-0.1914) = 21.9 minutes

We used the following calculation to demonstrate how this increase could be achieved activities equivalent to brisk walking (1.5 m/s^2) in females:
1440 x (0. 0579)/(1.5-0.1914) = 63.7 minutes

We used the following calculation to demonstrate how this increase could be achieved through light-intensity activities equivalent to 0.75 m/s^2 in females:
1440 x (0.0579)/(0.75-0.1914) = 149.3 minutes

We used the following calculation to demonstrate how this increase could be achieved through high-intensity activities equivalent to 4 m/s^2 in males:
1440 x (0.0696)/(4-0.2151) = 26.5 minutes

We used the following calculation to demonstrate how this increase could be achieved activities equivalent to brisk walking (1.5 m/s^2) in males:
1440 x (0.0696)/(1.5-0.2151) = 78.0 minutes

We used the following calculation to demonstrate how this increase could be achieved through light-intensity activities equivalent to 0.75 m/s^2 in males:
1440 x (0.0696)/(0.75-0.2151) = 187.4 minutes

References

1. Rowlands AV, Edwardson CL, Davies MJ, Khunti K, Harrington DM, Yates T. Beyond Cut Points: Accelerometer Metrics that Capture the Physical Activity Profile. Med Sci Sports Exerc. 2018;50(6):1323-32.
